# Radiation Interception, Chlorophyll Fluorescence and Senescence of Flag leaves in Winter Wheat under Supplemental Irrigation

**DOI:** 10.1038/s41598-017-07414-2

**Published:** 2017-08-10

**Authors:** Jianguo Man, Zhenwen Yu, Yu Shi

**Affiliations:** 1 National Key Laboratory of Crop Genetic Improvement, MOA Key Laboratory of Crop Ecophysiology and Farming System in the Middle Reaches of the Yangtze River, College of Plant Science and Technology, Huazhong Agricultural University, Wuhan, Hubei 430070 China; 2 Crop cultivation and farming scientists in the Key Laboratory of Crop Ecophysiology and Farming System, Ministry of Agriculture, College of Agronomy, Shandong Agricultural University, Tai`an, Shandong 271018 China

## Abstract

Water shortage threatens agricultural sustainability in China, effective water-saving technologies urgently need to be developed. In this study, five treatments were conducted: rainfed (W0), a local supplemental irrigation (SI) practice (W1), and three treatments in which soil water content was tested prior to SI, specifically at 0–20 (W2), 0–40 (W3) and 0–60 cm (W4) soil layers. Soil water consumption in W3 had no differ with W2 but was higher than W1 and W4. Crop evapotranspiration in W1, W3 and W4 treatments were higher than that in W2. W3 treatment had higher leaf area index than W1 and W4 at later grain filling stages. The mean photosynthetically active radiation capture ratio in W3, especially at 20, 40 and 60 cm plant heights, were significantly higher than those in W1, W2 and W4. The chlorophyll content index, actual photosynthetic activities, catalase and superoxide dismutase activities of flag leaves from W3 were the highest after the middle grain filling stages. W3 treatment obtained the highest grain yield (9169 kg ha^−1^) and water use efficiency (20.8 kg ha^−1^ mm^−1^) in the two seasons. These benefits likely accrued through created a suitable soil moisture environment in W3 treatment.

## Introduction

Winter wheat (*Triticum aestivum* L.) is one of the major crops in the Huang-Huai-Hai Plain of China. More than 60% of wheat produced in China is grown here^1^. The climate is a warm-temperate continental monsoon type with an annual average temperature range of 3.1 °C in the north to 16.8 °C in the south^2^. In this region, the total water consumption required by winter wheat is approximately 400–500 mm in a growing season, but only 150–180 mm of precipitation falls during this period^3^. Additional irrigation is thus required. Farmers using traditional practices in this region irrigate wheat crops with up to 310 mm of water, leading to the low water use efficiency (WUE)^4^. Therefore, effective water-saving technologies urgently need to be developed to maintain a high production of winter wheat.

In recent years, supplemental irrigation (SI) has been studied as a highly efficient practice with great potential for increasing agricultural production and improving livelihoods, which was widely used to improve crop yield and WUE^5^. Abourached *et al*. suggested that SI applied at heading and/or after heading could reduce water shortage stress during the grain-filling period and increase grain yield and WUE^6^. To obtain higher grain yield and WUE than the traditional irrigation practices, Wang *et al*. recommended two applications, at jointing and booting, with 75 mm each time, whereas Li *et al*. recommended two applications, at jointing and heading, with 60 mm each time, which was considered as one of the water-saving irrigation regimes in the Huang-Huai-Hai Plain of China^7–9^. However, most of the studied focused on fixed amounts of irrigation and, there are some limitations, as did not consider the effect of soil water conditions before irrigation (particularly those at different soil depths) on the irrigation amount, water consumption and winter wheat production. Therefore, irrigation practices based on the consideration of precipitation, soil water supply, and the physiological requirement of wheat grown in this region to increase water-saving in wheat production need to be developed.

The grain yield of a crop is dependent on the leaf area index (LAI), the ability of the canopy to intercept incoming radiation and photosynthesis of leaves^10^. Within an appropriate range, LAI increases with an increased supply of irrigation, and the greater the LAI, the greater is its photosynthetically active radiation (PAR) interception^11^. Li *et al*. showed that irrigating three times at jointing, heading and milking (for a total of 180 mm irrigation) changed the vertical distribution of LAI, significantly improving the PAR capture ratio and radiation use efficiency and increasing the winter wheat grain yield^9^. Chlorophyll fluorescence, an important component of plant photosynthesis, can be used as an indicator to evaluate yield performance and is particularly sensitive to water deficit^12, 13^. Maximum photochemical efficiency (Fv/Fm) and actual photochemical efficiency (ΦPSII) of photosystem II (PSII) decline with water stress^14, 15^. Photosynthetic activities (chlorophyll content and fluorescence) of plants are enhanced under moderate soil moisture with reductions under both severe water deficit and excessive water conditions^16^. Leaf photosynthesis declines during grain filling, when leaves start to senesce and the photosynthetic apparatus breaks down^17^. Leaf senescence is controlled by both developmental programs and environmental signals. The senescence of flag leaves can be exacerbated by water stress or water-logging^18, 19^. During grain filling, water stress accelerates leaf senescence in wheat by increasing the production of reactive oxygen species. Superoxide dismutase (SOD) and catalase are the major components of these antioxidant systems, which can protect cells from oxidative damage during periods of water stress conditions^20^. However, knowledge of how winter wheat LAI, radiation interception, chlorophyll content and fluorescence, and senescence of flag leaves respond to SI that is determined by measuring the moisture at different soil depths is relatively limited.

In this study, we adapted a method using SI to recharge soil water at critical developmental stages to determine the irrigation amount^21^, a target relative soil water content of 65% of the field water-holding capacity (FC, FC: the maximum hanging capillary water content that soil can be maintained) at jointing and 70% FC at anthesis in three soil layers (0–20, 0–40 and 0–60 cm) were designed. The objectives of this study were, therefore, (1) to investigate the soil water use and crop evapotranspiration of winter wheat to SI, (2) to determine the responses of the winter wheat LAI and PAR capture ratio to SI by measuring soil water content, and (3) to clarify the effect of SI by measuring soil water content on the chlorophyll content, chlorophyll fluorescence, and winter wheat flag leaves senescence.

## Results

### Irrigation, soil water consumption and crop evapotranspiration

**Figure 1:**
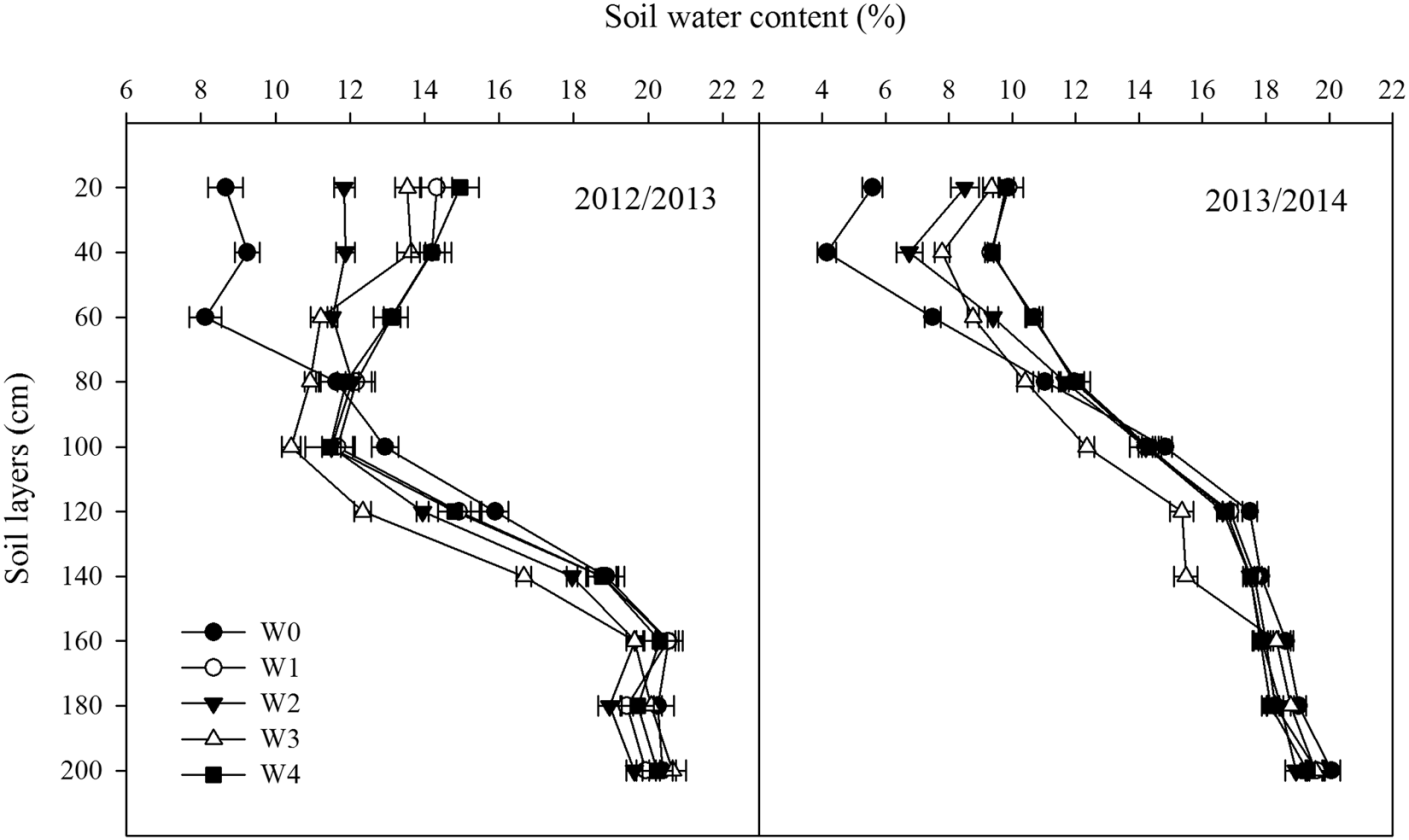
The soil water content at maturity of different treatments in 2012/2013 and 2013/2014 growing seasons. The horizontal bars represent standard error of the mean (n = 3).

The irrigation amount increased with increasing the measure depth from 20 cm (W2) to 60 cm (W4) (Table 1), the mean irrigation in W4 was 128.6 mm, which was higher by 33.4 mm and 73.0 mm than W3 and W2, respectively. The soil water content (SWC) in 0–200 cm soil layers at maturity in both seasons were presented at Fig. 1, the SWC in 60–140 cm soil layers of W3 was significantly lower than that in W1, W2 and W4, indicating that W3 had the highest soil water absorption in 60–140 cm soil layers among SI treatments.

**Table 1:** The target relative soil water content (θ_t_) and actual relative soil water content (θ_a_) in the supplemental irrigation treatments (W2, W3 and W4) after jointing and anthesis in 2012/2013 and 2013/2014 growing season, the amount of supplemental irrigation (CIR) is also indicated.

Treatments	Supplemental irrigation at jointing			Supplemental irrigation at anthesis			Total
	θ_t_(%)	θ_a_(%)	CIR (mm)	θ_t_(%)	θ_a_(%)	CIR(mm)	CIR(mm)
2012/2013							
W2	65(18.7)	64.7(18.6)	30.7	70(21.1)	71.1(20.4)	32.0	62.8
W3	65(17.8)	63.5(17.4)	49.6	70(19.2)	68.2(18.7)	51.2	100.8
W4	65(17.7)	63.5(17.3)	65.8	70(19.1)	67.9(18.5)	66.7	132.5
2013/2014							
W2	65(18.9)	64.1(18.6)	23.8	70(20.3)	68.2(19.8)	24.5	48.3
W3	65(18.5)	63.4(18.1)	44.0	70(20.0)	70.3(20.0)	45.5	89.6
W4	65(20.3)	64.1(17.9)	58.0	70(19.6)	72.8(20.4)	66.7	124.7

The treatments of W2, W3 and W4 were supplemental irrigation determined by measuring 0–20 cm, 0–40 cm and 0–60 cm soil layer moisture, respectively, and brought the soil moisture to 65% field water-holding capacity (FC) at jointing and 70% FC at anthesis.

The data in the bracket are the soil water content by weight-base, which is calculated as relative soil water content multiples FC.

**Figure 2:**
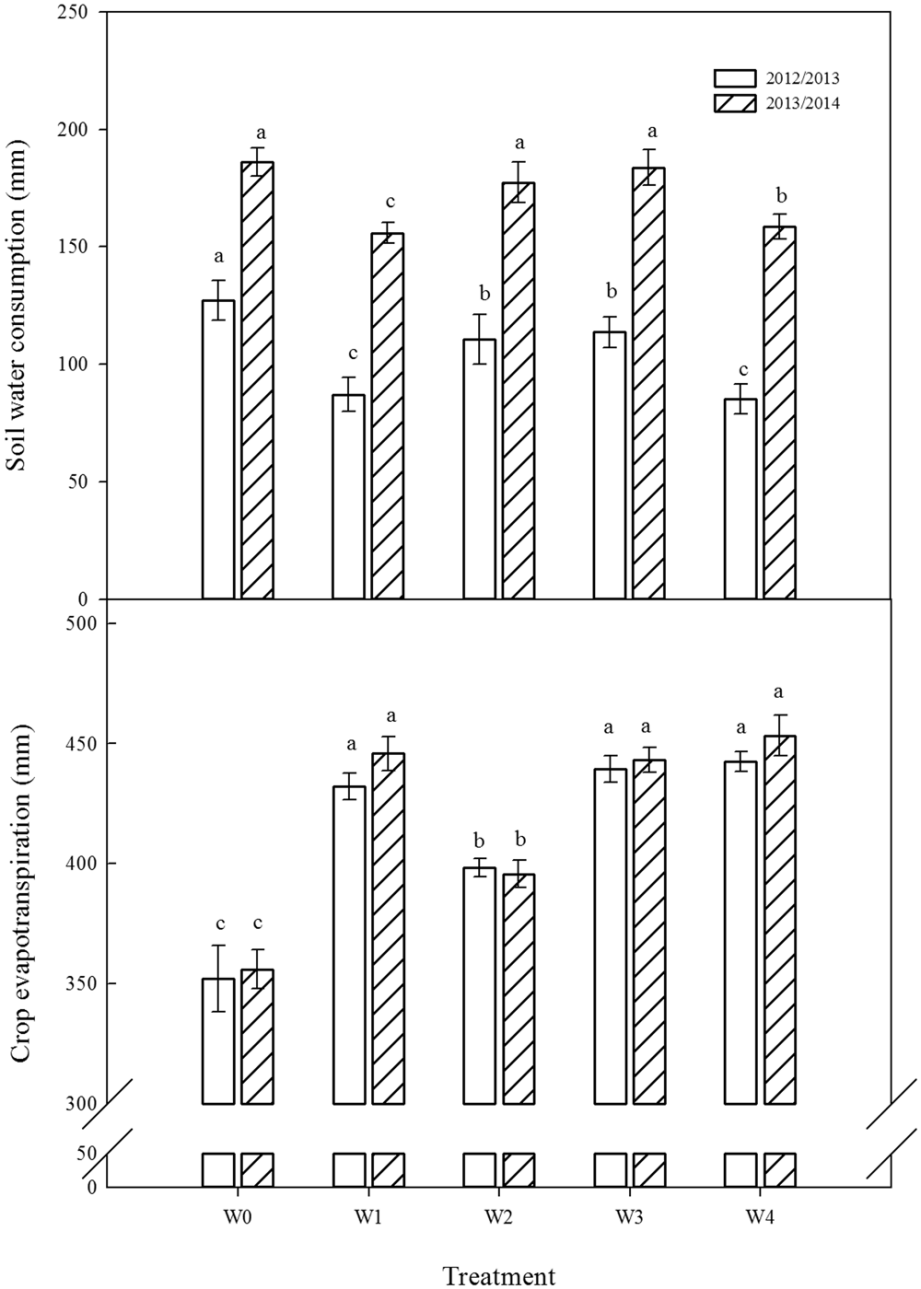
Responses of soil water consumption and crop evapotranspiration from 0–200 cm soil profile to different treatments in 2012/2013 and 2013/2014 growing seasons: rainfed (W0), a local supplemental irrigation practice with 60 mm of irrigation each at jointing and anthesis (W1), supplemental irrigation determined by measuring 0–20 cm (W2), 0–40 cm (W3), and 0–60 cm (W4) soil layers moisture and brought the soil moisture to 65% field capacity (FC) at jointing and 70% FC at anthesis. In the same growing season, the different letters in the figure are significant at the 0.05 level. Vertical bars are standard error of the mean (n = 3).

The soil water consumption (ΔW) from the W0 treatment was higher than that of the other treatments in 2012/2013 and 2013/2014 (Fig. 2). For the SI treatments, in 2012/2013, the highest ΔW was obtained from the W3 treatment, followed by the W2, the lowest ΔW was obtained in the W1 and W4 treatments. In 2013/2014, there was no significant difference in ΔW between W2 and W3 treatments, but the values were significantly higher than those from W1 and W4. The crop evapotranspiration (ET_c_) from the W1, W3 and W4 treatments did not differ in 2012/2013, but the ET_c_ in the W3 treatment was lower than that of the W1 and W4 treatments in 2013/2014. The W2 treatment had the lowest ET_c_ among the irrigation treatments. W0 had the lowest ET_c_ values in both growing seasons.

### Leaf area index

**Figure 3:**
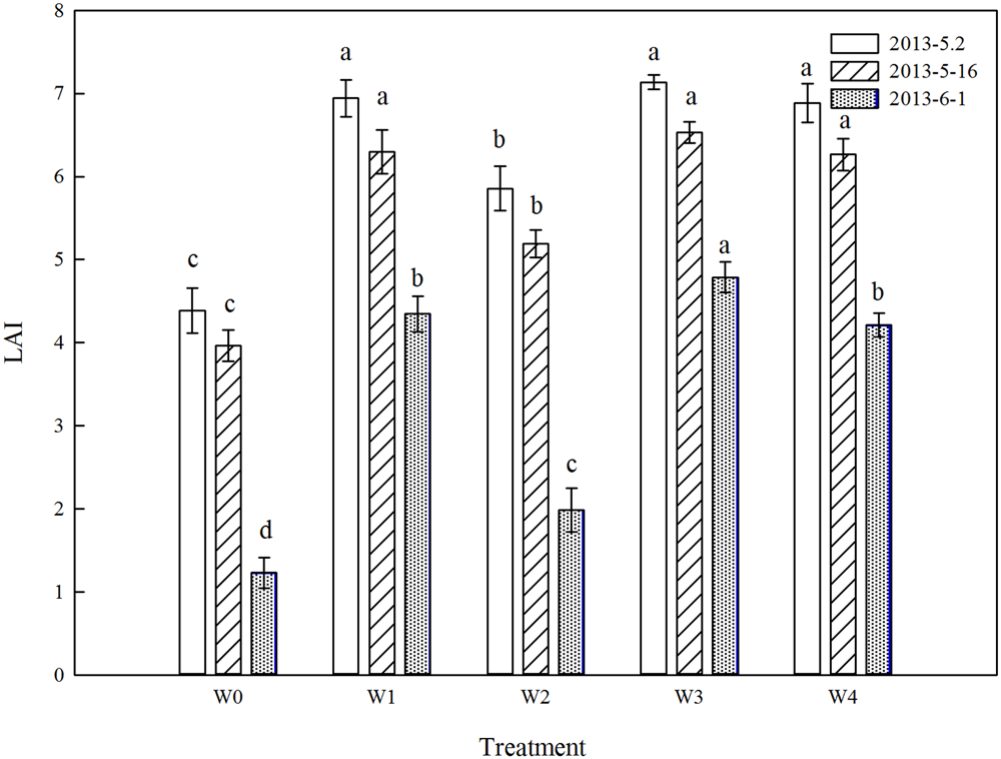
Changes of leaf area index (LAI) at May 2, May 16, and June 1 in 2013 of different treatments in 2012/2013 growing season: rainfed (W0), a local supplemental irrigation practice with 60 mm of irrigation each at jointing and anthesis (W1), supplemental irrigation determined by measuring 0–20 cm (W2), 0–40 cm (W3), and 0–60 cm (W4) soil layers moisture and brought the soil moisture to 65% field capacity (FC) at jointing and 70% FC at anthesis. At the same measuring day, the different letters in the figure are significant at the 0.05 level. Vertical bars are standard error of the mean (n = 3).

The LAI of plants from the W0 treatment was the lowest at May 2, May 16, and June 1 in 2013 (Fig. 3). For the SI treatments, LAI from the W3 treatment were higher than those from W1 and W4, and the differences were significant at June 1; the lowest LAI was obtained in the W2 treatment. Compared with the W3 treatment, the mean LAI of plants from the W1, W2 and W4 treatments were lower by 5.2%, 23.3% and 6.4%, respectively. The differences in LAI of plants from each treatment after anthesis in 2013/2014 were similar to those obtained in 2012/2013, therefore, the data are not shown.

### Radiation interception

**Figure 4:**
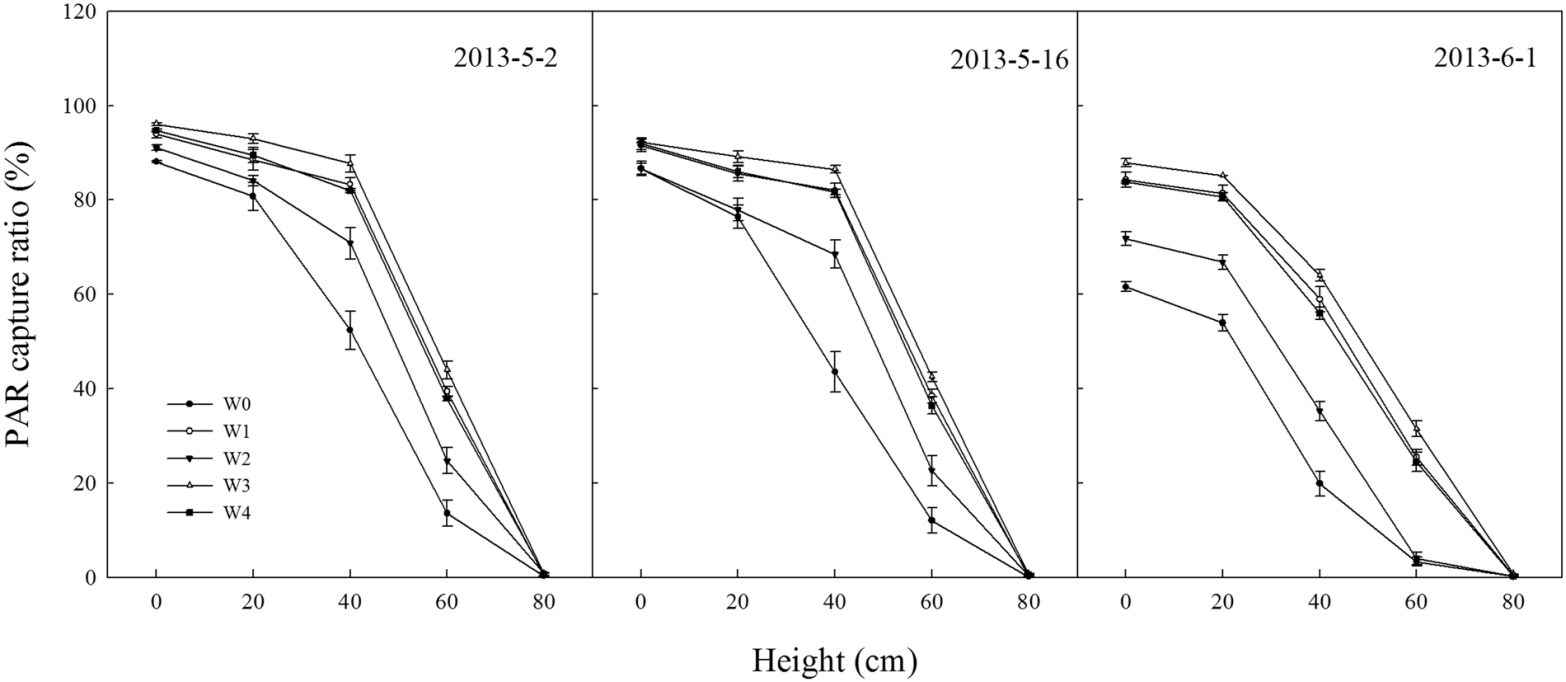
Changes of PAR capture ratio in different plant heights at May 2, May 16, and June 1 in 2013 of different treatments in 2012/2013 growing season: rainfed (W0), a local supplemental irrigation practice with 60 mm of irrigation each at jointing and anthesis (W1), supplemental irrigation determined by measuring 0–20 cm (W2), 0–40 cm (W3), and 0–60 cm (W4) soil layers moisture and brought the soil moisture to 65% field capacity (FC) at jointing and 70% FC at anthesis. The vertical bars represent standard error of the mean (n = 3).

The data of PAR reflection ratio, penetration ratio, and capture ratio in the winter wheat canopy during the grain filling stage between treatments in 2013/2014 were similar with those obtained in 2012/2013 (Table 2), which are not shown. The PAR capture ratio of plants from W3 was 92.1%, which was higher by 2.5%, 10.7% and 2.1% than that from W1, W2 and W4. The lowest PAR capture of plants was obtained in the W0 treatment. By contrast, the highest PAR penetration ratio of plants was obtained in the W0, followed by the W2, and then the W1 and W4 treatments. The lowest PAR penetration ratio was obtained in the W3 treatment. The PAR reflection ratios of plants in various treatments did not differ. The changes in the PAR capture ratio at different plant heights on May 2, May 16 and June 1, in 2013 are shown in Fig. 4. On May 2 and May 16, the PAR capture ratios at a height of 0 cm did not differ among the W1, W3 and W4 treatments, but these were higher than that from W2; the highest PAR capture ratios at heights of 20, 40 and 60 cm were obtained in W3, followed by W1 and W4, and then W2. On June 1, the PAR capture ratios at heights of 0, 20, 40 and 60 cm from W3 were the highest, followed by the W1 and W4, and the W2 treatment had the lowest values among the SI treatment. Compared to the SI treatments, the lowest PAR capture ratio were obtained in W0 at heights of 0, 20, 40 and 60 cm on the three measure days.

**Table 2:** Photosynthetically active radiation (PAR) capture ratio, penetration ratio, and reflection ratio in winter wheat canopy of each treatment (%)

Treatments	Capture ratio	Penetration ratio	Reflection ratio
W0	78.8d	18.3a	3.0a
W1	89.9b	7.1c	3.0a
W2	83.2c	14.3b	2.5a
W3	92.1a	5.3d	2.6a
W4	90.2ab	6.8c	3.0a

The data were the average values on May 1, May 16, and June 1 in 2013.

W0: rainfed, W1: a local supplemental irrigation practice at jointing and anthesis with 60 mm each time, W2, W3 and W4 are supplemental irrigation determined by measuring 0–20 cm, 0–40 cm, and 0–60 cm soil layer moisture, respectively, and brought the soil moisture to 65% FC at jointing and 70% FC at anthesis.

Within a column values followed by different letters differ significantly at the 0.05 level by the LSD test.

### Chlorophyll content and chlorophyll fluorescence

**Figure 5:**
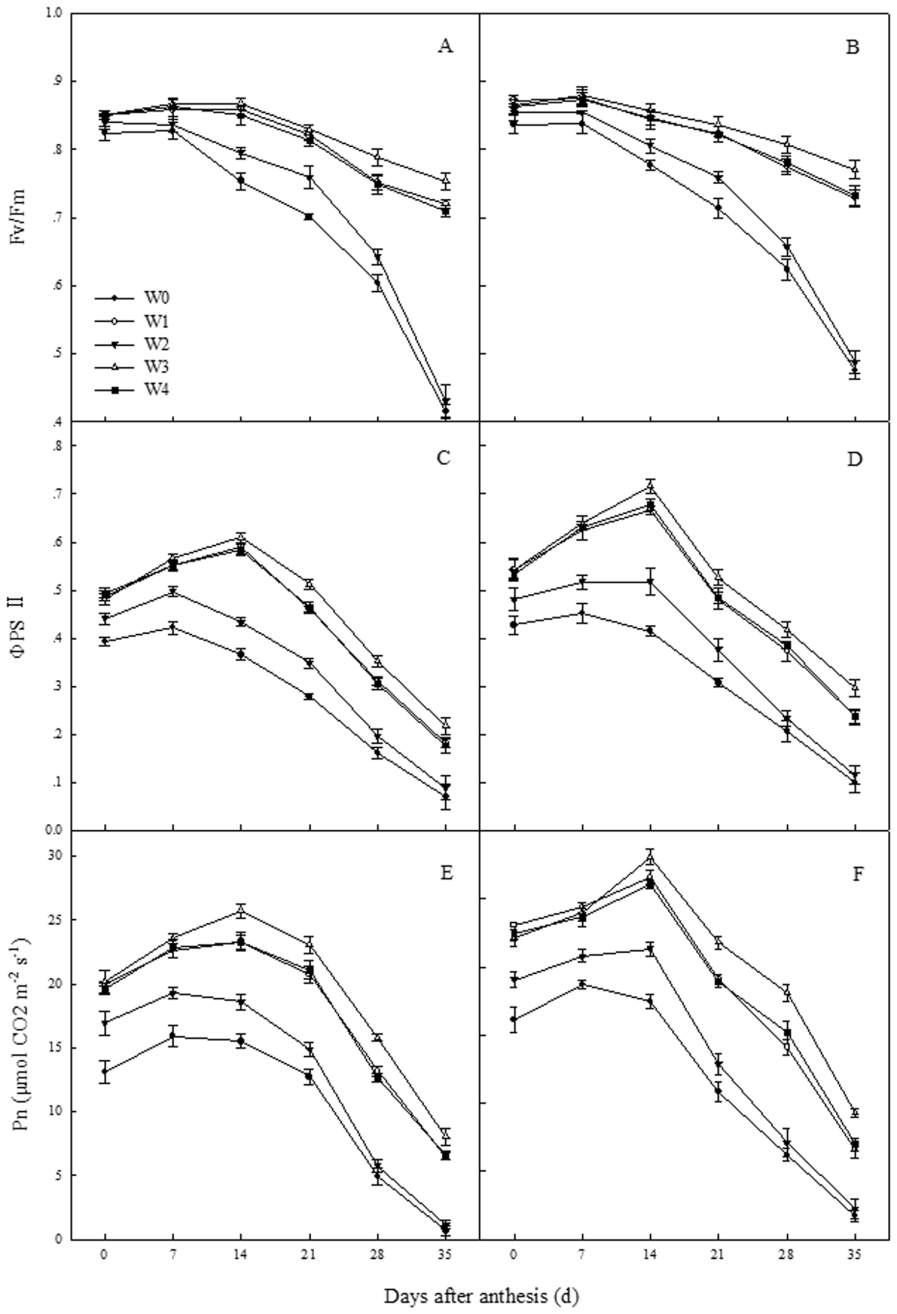
Changes of the Chlorophyll content index (CCI, **A** and **B**)maximum quantum yield of the PSII (Fv/Fm, **C** and **D**) and actual photochemical efficiency (ΦPSII, **E** and **F**) of different treatments in 2012/2013 (**A**, **C** and **E**) and 2013/2014 (**B**,**D** and **F**) growing seasons: rainfed (W0), a local supplemental irrigation practice with 60 mm of irrigation each at jointing and anthesis (W1), supplemental irrigation determined by measuring 0–20 cm (W2), 0–40 cm (W3), and 0–60 cm (W4) soil layers moisture and brought the soil moisture to 65% field capacity (FC) at jointing and 70% FC at anthesis. The vertical bars represent standard error of the mean (n = 3).

In 2012/2013, the CCI and F_v_/F_m_ of flag leaves from 0 to 21 days after anthesis (DAA) from W1, W3 and W4 treatment had no differ, but the values from 28 to 35 DAA from W3 were significantly higher than those from W1 and W4 (Fig. 5); there were no differ in the ΦPSII of flag leaves between W1, W3 and W4 treatments from 0 to 7 DAA, however, W3 had higher ΦPSII than those from W1 and W4 from 14 to 35 DAA. In 2013/2014, the CCI of flag leaves from 0 to 21 DAA from W1, W3 and W4 treatment had no differ, but the values from 28 to 35 DAA from W3 were significantly higher than those from W1 and W4; there were no differ in the F_v_/F_m_ and ΦPSII of flag leaves between W1, W3 and W4 treatments from 0 to 7 DAA, however, W3 had higher F_v_/F_m_ and ΦPSII than those from W1 and W4 from 14 to 35 DAA. The CCI, F_v_/F_m_ and ΦPSII of flag leaves from the W0 treatment were lowest during the grain filling stage in both growing seasons.

### Leaf senescence characteristics

**Figure 6:**
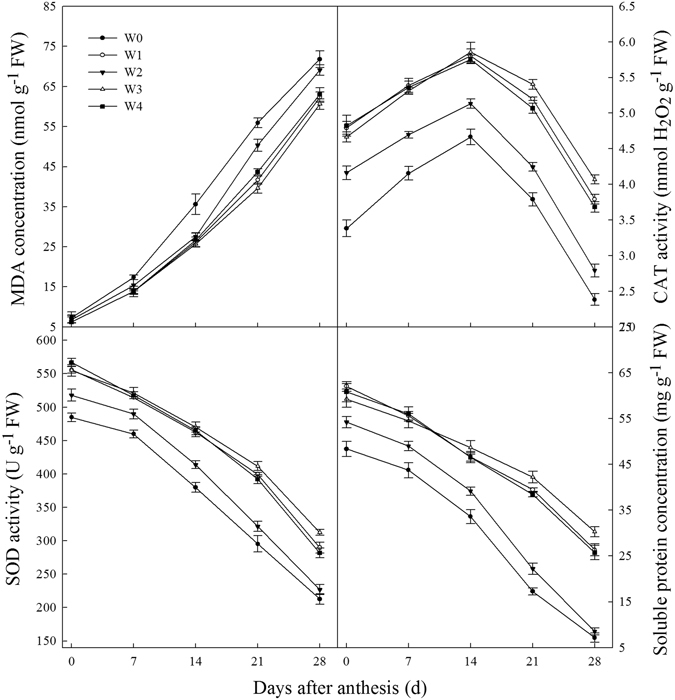
Changes of malondialdehyde (MDA) concentration, catalase (CAT) and superoxide dismutase (SOD) activities, and soluble protein concentration in flag leaves of wheat to different treatments in 2012/2013 growing seasons: rainfed (W0), a local supplemental irrigation practice with 60 mm of irrigation each at jointing and anthesis (W1), supplemental irrigation determined by measuring 0–20 cm (W2), 0–40 cm (W3), and 0–60 cm (W4) soil layers moisture and brought the soil moisture to 65% field capacity (FC) at jointing and 70% FC at anthesis. The vertical bars represent standard error of the mean (n = 3).

There were no significant differences among treatments in malondialdehyde (MDA) concentration of flag leaves at the beginning of grain filling in 2012/2013 growing season (Fig. 6). However, the highest MDA concentrations in flag leaves were obtained from the W0 treatment from 7 to 28 DAA. The MDA concentrations in flag leaves from W2 were significantly higher than those from W1, W3 and W4 from 21 to 28 DAA, but the values between W1, W3 or W4 did not differ. By contrast, the lowest catalase activities, SOD activities and soluble protein concentrations in flag leaves were obtained from the W0 treatment. The catalase, SOD activities and soluble protein concentrations in flag leaves from W2 were significantly lower than those from W1, W3 and W4 during the grain filling stage. The CAT, SOD activities and soluble protein concentrations in flag leaves between the W1 and W3 or W4 treatments from 0 to 14 DAA were not differ, but from 21 to 28 DAA, those parameters from the W3 were significantly higher than those from W1 and W4 treatments. The differences in MDA, catalase activities, SOD activities and soluble protein concentrations in flag leaves of plants from each treatment during grain filling stage in 2013/2014 were similar to those obtained in 2012/2013, therefore, the data is not shown.

### Grain yield, water use efficiency and harvest index

The grain yield, WUE and HI of different treatments in 2012/2013 and 2013/2014 growing seasons are shown in Table 3. The grain yield, WUE and HI from W3 were higher than those from W1 and W4, and the lowest values were from W2 among SI treatment in both growing seasons. The mean grain yield and WUE in the W3 treatment in both growing seasons were higher by 3.4% and 2.8% than those from W1 and higher by 4.4% and 6.0% than those from W4, respectively. Compared to the SI treatments, W0 had the lowest grain yield, WUE and HI in both growing seasons.

**Table 3:** The grain yield, water use efficiency (WUE), and harvest index (HI) in 2012/2013 and 2013/2014 growing seasons.

Year	Treatments	Grain yield (kg ha^−1^)	WUE (kg ha^−1^ mm^−1^)	HI (%)
2012/2013	W0	6123d	17.4d	44.2ab
	W1	8779b	20.3ab	43.9ab
	W2	7179c	18.0 cd	43.1b
	W3	9077ab	20.7a	45.1a
	W4	8701b	19.7b	44.2ab
2013/2014	W0	6407d	18.0 cd	41.3 cd
	W1	8952ab	20.1ab	41.7c
	W2	7367c	18.6c	40.1d
	W3	9260a	20.9a	43.7ab
	W4	8855b	19.5b	41.8c

W0: rainfed, W1: a local supplemental irrigation practice at jointing and anthesis with 60 mm each time, W2, W3 and W4 are supplemental irrigation determined by measuring 0–20 cm, 0–40 cm, and 0–60 cm soil layer moisture, respectively, and brought the soil moisture to 65% FC at jointing and 70% FC at anthesis.

Within a column values followed by different letters differ significantly at the 0.05 level by the LSD test.

## Discussion

Irrigation increases crop evapotranspiration, while soil water consumption has a negative relationship with irrigation^22^. In this study, the irrigation amount was determined by the water content of different soil layers, which was used in our previous studies^21, 23^. The mean irrigation amount from the W3 treatment was 95.2 mm, significantly higher than that of the W2 treatment but lower than that of the W1 and W4 treatments, the crop evapotranspiration from W3 was higher than that of W2 but lower that of W1 and W4, however, the mean soil water consumption from the W3 treatment was higher than that of the W1 and W4 treatments by 27.2 and 26.8 mm, respectively (Fig. 1). These results were also similar with Xue *et al*. and Qiu *et al*., who demonstrated that irrigation increased from 135.0 mm to 265.0 mm and ET_c_ increased by 96.6 mm, whereas the soil water consumption dropped by 33.4 mm^24, 25^.

Soil water use by plant is associating with root growth and development. Studies found that reasonable irrigation regimes with lower water stress could facilitate root growth, especially in deep soil layers, which is conductive to water absorption from soil^7, 24^. Xue *et al*. showed that the water uptake rate in 0–100 cm soil layers was significantly lower in rainfed than in irrigation treatments because of low root density^26^. Li *et al*. reported that irrigation of 120 mm only at jointing results in the highest root length density, leading to the highest soil water consumption in 0–160 cm soil layers^9^. In this study, the irrigation amount in W3 ranges from 89.6 mm to 100.8 mm, which was significantly higher than that in W2 but lower than that in W4 (Table 1). However, the soil water use in W3 was the highest (Fig. 2), particularly in the 60–140 cm soil layers (Fig. 1), which are likely because that the irrigation determined by measuring the moisture in 0–40 cm soil layer facilitate root growth in the 60–140 cm soil layer, improve water use from soil^27^.

Suitable irrigation amount could improve grain yield and water use efficiency^28, 29^. Karam *et al*. reported that irrigation with 50% of the full SI (based on the SWC in 0–90 cm soil layers) achieved 340 kg ha^−1^ greater yield than that of the 100% SI treatment^28^. While, Boutraa *et al*. concluded that plants grown under 80% FC in the 0–120 cm soil layer have the highest grain yield and WUE^29^. Here, our results showed that the highest grain yield, WUE and HI were obtained in the W3 treatment, with mean values of 9169 kg ha^−1^, 20.8 kg ha^−1^ mm^−1^ and 44.5%, respectively, in both growing seasons, which indicate that the appropriate irrigation amount obtained by measuring the 0–40 cm soil layer moisture created a suitable soil environment (approximately 65% FC and 70% FC after SI at jointing and anthesis, respectively), which increased the use of soil water and improved the grain yield and WUE.

Leaf area index and PAR are the main factors determining crop growth in wheat, which is significantly affected by irrigation^9, 30^. Ram *et al*. reported that LAI increased with increased irrigation in wheat, but the difference was not significant as more than 225 mm of irrigation^11^, we obtained the same results in this study, and we also found that the LAI dropped at later stages of winter wheat as the total irrigation amount over 95.2 mm. The LAI was one of the most important factors on affecting the amount of incoming PAR absorbed by the canopy, and the greater the crop LAI, the greater is its PAR interception^31^. In this study, the LAI from the W3 treatment was higher than that of the W1, W2 and W4 treatments, the PAR capture ratio was also higher than those of the W1 and W4 treatments, especially at later grain filling stages, the results indicating that suitable soil water conditions could improve the LAI and PAR at these stages. We also found that the PAR capture ratio at heights from 20 cm to 60 cm in the W3 treatment was higher than that of the other treatments during the grain filling stage (Fig. 3), which indicates that the PAR capture ratio in the upper canopy of winter wheat had a large contribution to the final grain yield.

Both Saeidi *et al*. and Mu *et al*. reported that more than 70% of winter wheat yield is produced by photosynthesis in spikes and leaves after heading^32, 33^. Photosynthesis is directly affected by chlorophyll content and chlorophyll fluorescence, which decreased significantly in water deficit conditions and resulted in a reduction in photosynthetic capacity^34, 35^. In this study, the CCI, F_v_/F_m_, and ΦPSII of flag leaves from the W3 treatment were higher than those from W1, W2 and W4 treatments after 21 DAA (Fig. 5). This result indicated that SI applied at jointing and anthesis by measuring the soil moisture of the 0–40 cm layers created a suitable soil environment, which improved the photosynthetic capacity at the middle and later grain filling stages.

Loss of leaf viability during senescence closely links the duration of photosynthetically active leaf area and grain yield in wheat^36^. Leaf photosynthesis declines during grain filling, when leaves start to senesce and the photosynthetic apparatus disassembles rapidly within chloroplasts^17^. This senescence-associated decline in photosynthetic capacity of leaves can be exacerbated by water stress or waterlogging^19, 37^. Here, our results also found that the MDA concentrations in W0 flag leaves were higher than those in flag leaves from SI treatments. On the contrary, the lowest SOD and catalase activities were observed in the W0 treatment. Saeedipour and Moradi also observed that irrigation with 50% FC after anthesis enhanced the senescence by accelerating loss of leaf chlorophyll and soluble proteins^38^. In this study, there were no significant differences in MDA in flag leaves between the W1 and W3 or W4 treatments, but the SOD and catalase activities and soluble protein concentrations in flag leaves from the W3 treatment were significantly higher than those from the W1 and W4 treatments after 14 DAA (Fig. 4). These findings are likely the causes of the high chlorophyll content and fluorescence in flag leaves from the W3 treatment at the middle and later stages of grain filling, which are beneficial to wheat production^18, 36^.

## Conclusions

Supplemental irrigation amount (SI) at jointing and anthesis, determined by measuring the soil moisture of the 0–40 cm layer (W3), enhanced the soil water consumption, increased the leaf area index at the later grain filling stages, and improved the photosynthetically active radiation capture ratio, especially at plant heights from 20 to 60 cm. The highest chlorophyll content index, maximum quantum yield of the PSII, and actual photochemical efficiency at the middle and later stages of grain filling were obtained in W3, which were likely because of the high superoxide dismutase and catalase activities and soluble protein concentrations of flag leaves during the stages, and finally increased the grain yield and water use efficiency. It is hypothesized that these benefits accrued through created a suitable soil moisture environment in W3 treatment.

## Materials and Methods

### Experimental site

**Figure 7:**
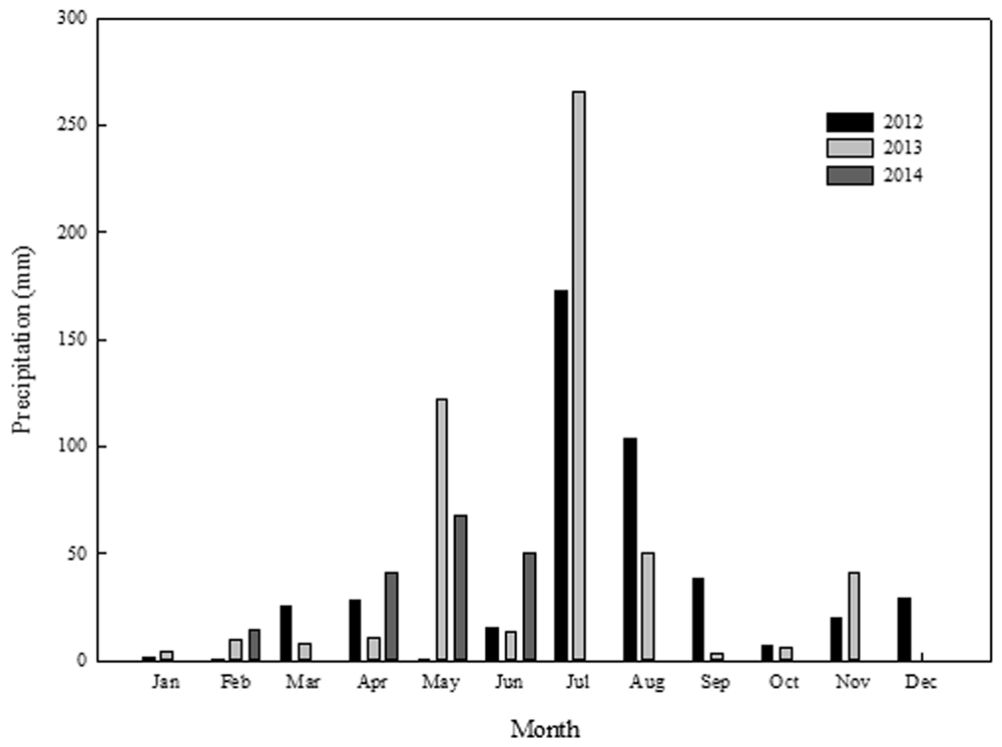
Monthly precipitation in 2012, 2013 and 2014. Precipitations from July to December were not measured in 2014. Data was obtaind by the Jining Meteorological Bureau (http://www.jnqxj.gov.cn/).

Field experiments were conducted from October 2012 to June 2014 in Shijiawangzi Village, Yanzhou, Shandong Province, China (116°41′E, 35°42′N). This village is located in the centre of the Huang-Huai-Hai Plain, and its environment is typical and representative of the plain. The area has a warm temperate semi-humid continental monsoon climate, the annual temperature, accumulated sunshine and precipitation ranges from −5.3 to 32.6°C, 1686 to 2734 h and 353.5 to 1183.7 mm, respectively. The precipitation amounts in 2012, 2013 and 2014 were 509.1, 534.5 and 530.1 mm (225.0 and 170.0 mm during winter wheat growing seasons in 2012/2013 and 2013/2014, respectively), respectively, which were the dry years, and the frequencies of the similar years are 36% according to the classification of AQSIQ and SAC^39^. The monthly precipitation in 2012, 2013 and 2014 were presented in Fig. 7. All of the meteorological data were obtained by the Local Meteorological Bureau of Jining, China (http://www.jnqxj.gov.cn/). The soil type is clay loam soil with a composition of 29.6% clay, 37.3% silt, and 33.1% sand as determined by the classification of soil in the USDA taxonomy^40^. The altitude of this area is 33.2 m.

The organic matter, total nitrogen, available phosphorus and available potassium in the topsoil (0–20 cm) of the experimental plots were 15.9 g kg^−1^, 1.2 g kg^−1^, 30.9 mg kg^−1^, and 114.5 mg kg^−1^, respectively, according to potassium dichromate colorimetric method, the Kjeldahl method, the sodium bicarbonate method and the ammonium acetate method, respectively. The soil dry bulk density, is defined as the ratio of dry soil weight to bulk soil volume was measured by cutting ring method according to MOA^41^, the equation is

1 $${{\rm{\gamma }}}_{{\rm{bd}}}={\rm{M}}/{\rm{V}},$$

where, γ_bd_ (g cm^−3^) is the soil bulk density; M (g) is the dry soil weight in the cutting ring; V (cm^3^) is the volume of the cutting ring, it is 100 cm^3^ in this study.

Field water holding capacity (FC) is the maximum hanging capillary water content that soil can be maintained, which was measured by cutting ring method according to MOA^42^, the equation is

2 $${\rm{FC}}=({{\rm{m}}}_{1}-{{\rm{m}}}_{2})\times 100/({{\rm{m}}}_{2}-{{\rm{m}}}_{0})$$

where, FC (%) is the field water-holding capacity; m_1_ (g) is the sum of soil weight after 8 hours of water draining before drying and the aluminium box weight; m_2_ is the sum of undisturbed soil dry weight after drying and the aluminium box weight; m_0_ is the weight of the aluminium box.

The values of soil bulk density, field water-holding capacity, and soil water content (SWC) before sowing of the top 0–200 cm of the soil (in 20-cm increments) are shown in Table 4, the SWC at maturity in different treatments are shown in Fig. 1. The groundwater depth is 25 m.

**Table 4:** Soil bulk density and soil water in the top 0–200 cm of the soil (in 20-cm increments) of the experimental field.

Soil layers (cm)	Soil bulk density (g cm^−3^)		Field water holding capacity (%)		Soil water content one day before sowing (%)	
	2012/2013	2013/2014	2012/2013	2013/2014	2012/2013	2013/2014
0–20	1.4	1.4	28.7	29.0	17.5	19.8
20–40	1.4	1.4	26.2	28.0	16.1	19.3
40–60	1.5	1.4	27.0	27.0	14.3	20.3
60–80	1.5	1.5	26.0	26.0	16.3	18.5
80–100	1.5	1.5	25.4	26.0	18.7	19.3
100–120	1.5	1.5	25.9	25.3	19.5	20.4
120–140	1.5	1.5	25.1	25.0	21.4	20.1
140–160	1.5	1.5	24.8	24.6	21.8	20.8
160–180	1.6	1.6	23.3	24.1	21.6	20.7
180–200	1.6	1.5	23.8	24.0	21.9	21.2

### Experimental design

Five treatments were designed: a rainfed (W0) treatment with no irrigation, a local SI practice treatment (W1, 60 mm of irrigation each at jointing and anthesis), and three treatments in which soil layers at specified depth were measured for SWC prior to SI: 0–20 cm (W2), 0–40 cm (W3), and 0–60 cm (W4). SI brought the mean SWC in each measured soil layers to 65% FC at jointing (Z31, first node detectable) and 70% of FC at anthesis (Z61, beginning of anthesis)^43^.

Three soil samples were collected using a soil corer (length 20.0 cm and diameter 5.0 cm) down to 20, 40, and 60 cm depth at the middle part in each plot of W2, W3, and W4 treatments, respectively. The SWC (gravimetric water content) of each treatment (average SWC of 0–20 cm in W2, 0–40 cm in W3, and 0–60 cm in W4 treatment, respectively) was determined using the oven-drying method^44^.

The amount of SI matched the crop irrigation requirement (CIR), which was calculated from the relative soil water content in the corresponding soil layers. The CIR was calculated using the following equation as described by Jalilian *et al*.^45^.

3 $${\rm{CIR}}=10{{\rm{\gamma }}}_{{\rm{bd}}}\times {D}_{{\rm{h}}}\times ({{\rm{SWC}}}_{{\rm{t}}}-{{\rm{SWC}}}_{{\rm{n}}})$$

where CIR (mm) is the amount of SI; γ_bd_ (g cm^−3^) is the soil bulk density; D_h_ (cm) is the depth of the soil layer; SWC_t_ (%) is the target soil water content after SI; SWC_n_ (%) is the soil water content before irrigation. SWC_t_ was calculated use equation (4):

4 $${{\rm{SWC}}}_{{\rm{t}}}={\rm{FC}}\times {{\rm{SWC}}}_{{\rm{tr}}}$$

where FC (%) is field water-holding capacity; SWC_tr_ (%) is the target relative soil water content (it was 65% at jointing and 70% at anthesis in this study). Uniform flood irrigation method was used, and a flow meter **(**accuracy: 0.001 m^3^, type: N15, linyi-mingquan Inc., China) was used to measure the amount of water applied. The relative SWC and SWC before and after irrigation, and the CIR for different treatments are shown in Table 1.

The experiment followed a randomised scheme, and all treatments were replicated three times. Each experimental plot was 4 × 4 m in size, and a 2.0-m-wide unirrigated zone was maintained between adjacent plots to minimize the effects of adjacent treatments.

### Crop management

All plots were supplied with 240 kg N ha^−1^, 150 kg P_2_O_5_ ha^−1^ and 150 kg K_2_O ha^−1^. All P and K fertiliser and half the N fertiliser were applied pre-sowing, and the remaining N fertiliser was topdressed at the jointing stage. The high-yielding wheat (*Triticum aestivum* L.) cultivar Jimai22 was used in the experiments. Wheat seeds were sown at a density of 180 plants m^−2^ on October 10, 2012, and October 9, 2013. Wheat seedling shoots ceased growth at the beginning of December and started to grow again at the end of February. During this period, the average daily temperature was below 0 °C. Wheat plants were harvested on June 12, 2013, and June 6, 2014.

### Crop water use

Crop evapotranspiration (ET_c_) was calculated using the soil water balance equation ^46^ for the growing season as:

5 $$E{T}_{c}={\rm{P}}+{\rm{CIR}}+{\rm{\Delta }}{\rm{W}},$$

where ET_c_ (mm) is the total crop evapotranspiration during a growing season; P (mm) is the precipitation; CIR (mm) is the amount of SI; ΔW (mm) is the soil water consumption, which was defined as the difference of soil water storage between sowing and harvesting, the equations are:

6 $${\rm{\Delta }}W={{\rm{S}}}_{{\rm{s}}}-{{\rm{S}}}_{{\rm{h}},}$$

7 $${{\rm{S}}}_{{\rm{i}}}=10{{\rm{\gamma }}}_{{\rm{bd}}}\times {{\rm{D}}}_{{\rm{h}}}\times {{\rm{SWC}}}_{{\rm{i}}}$$

Where ΔW (mm) is the soil water consumption; S_i_ (mm) is soil water storage (S_s_ and S_h_ are the soil water storage in 0-200 cm soil layers at sowing and harvesting, respectively); γ_bd_ (g cm^−3^) is the soil bulk density; D_h_ (cm) is the depth of the soil layer (D_h_ = 200 cm in this study); SWC_i_ (%) is the SWC on a weight-basis at sowing and harvesting, respectively.

No account was taken of capillary rise, runoff and drainage. When the groundwater table is lower than 2.5 m below the soil surface, as it is at the experimental site, the capillary rising of groundwater is negligible^47^; runoff can be ignored because of the terrier around the border in the North China Plain, including this experimental site^46^; the drainage is very little in this study, we assumed that the irrigation and precipitation was absorbed by winter wheat completely, therefore, the drainage was ignored here^21, 23^.

The water use efficiency of winter wheat was calculated using the method described by Wang *et al*.^48^, the equation is:

8 $${\rm{WUE}}={\rm{Y}}/{{\rm{ET}}}_{{\rm{c}}}$$

where WUE (kg ha^−1^ mm^−1^) is the water use efficiency for grain yield; Y (kg ha^−1^) is the grain yield; ET_c_ (mm) is the total crop evapotranspiration (water consumption) over the winter wheat growing season.

The harvest index (HI) is the grain yield over total above-ground biomass at maturity.

### Leaf area index and radiation interception

Green area of leaves from 30 plants in each plot was measured on May 2, May 16, and June 1 in 2013 using a leaf area meter (Winfolia Analysis System, Regent Instruments Inc., Canada), and the green leaves were scanned through the leaf area meter, the total leaf area were recorded, LAI is the leaf area for the 1-m^2^.

The photosynthetically active radiation interception was measured on typical sunny days (May 2, May 16, and June 1 in 2013) at heights of 0, 20, 40, 60, and 80 cm above the ground using the AccuPAR Ceptometer, model LP-80 (Decagon Devices, Inc. USA). The data were acquired using a 0.87-m linear sensor placed at the middle of wheat inter-rows parallel to wheat rows and at the vertical direction of wheat rows, respectively^49^. The PAR capture ratio was calculated as the ratio of the difference between incident and transmitted radiation to incident radiation. The PAR penetration ratio was calculated as the ratio of transmitted radiation to incident radiation, and the PAR reflection ratio was calculated as the ratio of PAR reflection measured at 50 cm above the canopy to incident radiation. These values were obtained from instantaneous measurements taken from 11:00 to 13:00 on sunny days^50^.

### Chlorophyll content and chlorophyll fluorescence

Flag leaf chlorophyll content index was measured by the CCM-200 Chlorophyll Content Meter (Opti-Science, Inc. USA.) on 10 flag leaves from each experimental plot. The fluorescence parameters (CFP) of flag leaves on which CCI were measured were determined using a pulse-modulated fluorimeter (FMS-2, Hansatech, UK). The minimum and maximum fluorescence (F_o_ and F_m_) were determined after a full-dark adaptation for 30 min. Steady state fluorescence (F_s_) was determined under actinic light. A saturating light pulse was applied to obtain F_m_ after each actinic light episode. The F_v_/F_m_ and ΦPSII were calculated according to Mu *et al*.^34^. and Zivcak *et al*.^12^. Measurements were made between 9:30 and 11:00 on days with full sunlight at seven-day intervals from anthesis to 35 DAA.

### Biochemical assays on flag leaves

Flag leaves from each experimental plot were sampled at seven-day intervals from anthesis to 28 DAA. At each sampling date, 20 flag leaves from each plot were detached, immediately submerged in liquid nitrogen, and then stored at −80 °C until biochemical assays were performed. The whole flag leaf (including the leaves and veins) was used to measure catalase and SOD activities, MDA and soluble protein concentrations in the leaf.

Catalase and SOD activities were extracted from flag leaves by grinding 2 g of leaf tissue in 5 ml extraction buffer (0.1 M phosphate, pH 7.5, containing 1.5 mM EDTA and 1 mM ascorbic acid) at 0 °C. The mixture was then centrifuged at 13000 × g for 20 min, and enzyme assays were performed on the supernatant^51^.

Catalase activity was assayed by measuring the initial rate of H_2_O_2_ disappearance^52^. A 3-ml reaction mixture contained 0.1 M sodium phosphate buffer (pH 7.0), 2 mM H_2_O_2_ and 0.1 ml of crude extract. The breakdown of H_2_O_2_ was followed by measuring the absorbance change at 240 nm, and enzyme activity was calculated using the extinction coefficient for H_2_O_2_ (40 mM cm^−1^ at 240 nm) according to Wang *et al*.^23^.

Superoxide dismutase activity was assayed by measuring inhibition of the photoreduction of nitro blue tetrazolium (NBT) following the method of Giannopolitis and Ries^53^. A 3-ml reaction mixture contained 50 μM NBT, 13 mM methionine, 75 μM NBT chloride, 0.1 mM EDTA, 50 mM phosphate buffer (pH 7.8), 50 mM sodium carbonate, and 0.1 ml crude extract. Test tubes containing this reaction mixture were placed under a light bank (15 fluorescent lamps) delivering 78 μmol m^−2^ s^−1^ for 15 min. Absorbance was determined at 560 nm using a spectrophotometer (Hitachi U-1100, Tokyo, Japan). One unit of SOD activity was defined as the amount of enzyme that inhibited NBT photoreduction by 50%.

Malondialdehyde concentrations of flag leaves were assayed according to Quan *et al*.^54^. MDA concentration was expressed as nmol g^−1^ fresh weight (FW).

Soluble protein concentrations of flag leaves were measured according to the Coomassie brilliant blue G250 method described by Read and Northcote^55^. Protein concentration was expressed as mg g^−1^ FW.

### Statistical analysis

Statistical analysis employed standard analysis of variance (ANOVA) using SPSS 13.0 software (SPSS Inc., Chicago, IL, USA.). The least significant difference (LSD) method was used to determine whether treatment means differed. The probability level for determination of significance was 0.05.
